# Fibulin-1 is required for microvascular integrity in the developing brain and retina

**DOI:** 10.1016/j.ydbio.2026.07.017

**Published:** 2026-08-01

**Authors:** Xuanyu Chen, Harshit Singhania, Syed Adeel H. Zaidi, Ishita Tandon, Roger I. Grant, Ruth B. Caldwell, Marion A. Cooley

**Affiliations:** a Department of Neuroscience and Regenerative Medicine, Medical College of Georgia, Augusta University, Augusta, GA, 30912, USA; b Department of Oral Biology, Dental College of Georgia, Augusta University, Augusta, GA, 30912, USA; c Vascular Biology Center, Medical College of Georgia, Augusta University, Augusta, GA, 30912, USA; d Culver Vision Discovery Institute, Medical College of Georgia, Augusta University, Augusta, GA, 30912, USA; e Department of Medicine, Medical College of Georgia, Augusta University, Augusta, GA, 30912, USA; f Immunology Center of Georgia, Medical College of Georgia, Augusta University, Augusta, GA, 30912, USA; g Department of Regenerative Medicine and Cell Biology, Medical University of South Carolina, Charleston, SC, 29425, USA; h Department of Cellular Biology and Anatomy, Medical College of Georgia, Augusta University, Augusta, GA, 30912, USA

**Keywords:** Glomeruloid vascular abnormalities, Retina, Intracerebral hemorrhage, Intraretinal hemorrhage, Tortuous capillaries, Familial exudative vitreoretinopathy

## Abstract

Vascularization of the central nervous system and the postnatal retina proceeds through angiogenic growth that relies in part on the basement membrane (BM) to support endothelial cell-cell interactions and maintain barrier integrity during vessel formation. In addition to the core structural components of the BM, matricellular proteins are present as minor constituents, however, their contributions to endothelial cohesion and barrier stability during angiogenesis remain poorly defined. Here, we identify the matricellular protein Fibulin-1 (Fbln1), a regulator of cell-matrix interactions, as an essential factor during mid-gestation that prevents the formation of dilated, tortuous capillaries that give rise to glomeruloid vascular lesions and intracerebral hemorrhage. Fbln1-deficient brains exhibit vascular malformations characterized by reduced type IV collagen deposition and diminished CD31 localization at endothelial junctions. Although overall pericyte coverage across the vasculature is maintained, the glomeruloid lesions lack pericyte investment. In the developing retina, loss of Fbln1 similarly results in hemorrhage and tortuous capillaries with glomeruloid vascular abnormalities that disrupt the formation of both the superficial and deep vascular plexuses. Together, these findings demonstrate that Fbln1 is a critical determinant of brain and retinal vascular development and is required to preserve endothelial barrier integrity during angiogenesis.

## 1. Introduction

Angiogenesis is the primary mechanism by which the central nervous system (CNS) becomes vascularized during embryogenesis. In the mouse, between embryonic day 8.5 and 10, endothelial cells (ECs) derived from mesoderm assemble around the neural tube to form the perineural vascular plexus (PNVP), the precursor of the CNS vasculature (Puelles et al., 2019; Zhang et al., 2024). ECs from this plexus then invade the neural tube through VEGF (vascular endothelial growth factor) guided sprouting to generate radial vessels that extend toward the ventricular surface. Subsequent branching of these vessels gives rise to the periventricular vascular plexus (PVP). Once this plexus is established, CNS vascular development proceeds through coordinated phases of vascular expansion, remodeling, and endothelial barrier maturation. (Puelles et al., 2019; Hogan et al., 2004; Darland et al., 2011; Fantin et al., 2013).

During this process, ECs interact closely with the basement membrane (BM), a specialized extracellular matrix (ECM) produced by ECs, pericytes, and astrocytes. The vascular BM is built from core structural proteins, including laminins (particularly α2/α4/α5 with β1 and ɣ1) and type IV collagen (primarily the α1α1α2 heterotrimer formed by COL4A1/COL4A2). These networks are stabilized by nidogens and heparan sulfate proteoglycans such as perlecan and agrin, which together maintain BM integrity (Thomsen et al., 2017; Hallmann et al., 2020). Genetic studies underscore the essential role of core BM components in CNS angiogenesis. Inactivating mutations in EPHB4 and RASA1, which cause Capillary Malformation-Arteriovenous Malformation (CM-AVM) syndrome in humans, lead to reduced type IV collagen deposition and increased susceptibility to intracerebral hemorrhage (Palermo et al., 2025; Chen et al., 2022). Similarly, loss of laminin α4 results in intracerebral hemorrhage, whereas deletion of laminin α2 or laminin ɣ1 compromises endothelial barrier integrity (Thyboll et al., 2002; Menezes et al., 2014; Yao et al., 2014). Beyond these core structural components, BM associated matricellular proteins, often considered minor constituents, also play indispensable roles in CNS vascular development. Through interactions with core BM proteins, these regulators modulate VEGF signaling, integrin dependent EC migration, and endothelial cell-cell interactions (Marchand et al., 2019; Eun et al., 2024; Chintala et al., 2015; Mongiat et al., 2016; Moon et al., 2020; Cydzik et al., 2015; Kaur et al., 2010). Despite these research advances, the functions of many matricellular proteins in CNS angiogenesis remain incompletely defined.

Fibulin-1 (Fbln1) is a secreted matricellular protein required for embryonic development. Mice lacking Fbln1 exhibit perinatal lethality with cardiac and pulmonary defects and pronounced hemorrhages in the telencephalon and spinal cord (Argraves et al., 1990; Cooley et al., 2008; Ito et al., 2020; Kostka et al., 2001; Harikrishnan et al., 2015). Although Fbln1 binds fibrinogen and was previously proposed to influence coagulation, mice lacking Fbln1 display normal clotting, platelet aggregation, and bleeding times, indicating that the hemorrhages do not arise from defects in hemostasis. The underlying cause of these hemorrhages remains poorly defined, however, ultrastructural analyses reveal microvascular abnormalities, including irregular capillary morphology and aberrant endothelial cytoplasmic projections that partially obstruct vessel lumens, while large-caliber vessels remain largely unaffected (Kostka et al., 2001; Tran et al., 1995). These findings suggest that Fbln1 contributes to microvascular stability by regulating endothelial cell-matrix interactions.

Fbln1 binds to several ECM proteins involved in angiogenesis, including ADAMTS1 (Lee et al., 2005; Lambert et al., 2020), the CCN family members CCN2 and CCN3 (Moon et al., 2020; Lin et al., 2003; Pi et al., 2012; Perbal et al., 1999), and Fibronectin (Fn) (Stenzel et al., 2011; Tran et al., 1997; Turner et al., 2017). Although these binding interactions suggest potential roles in endothelial regulation, their functional significance in vivo remains unclear. In some cell types, Fbln1 inhibits migration in a Fn-dependent manner through attenuation of ERK1/2 signaling, but EC such as HUVECs do not show this response (Twal et al., 2001). In contrast, ADAMTS1 together with Syndecan- 4 is reported to regulate Fbln1 synthesis and matrix incorporation, to suppresses EC migration and promotes α5 integrin recycling (Lambert et al., 2020). These findings point to a context-dependent mechanism by which Fbln1 can influence endothelial behavior during angiogenesis.

This study investigates the role of Fbln1 in CNS vascular development using germline and inducible Fbln1-deficient mouse models. Angiogenic patterning and vascular remodeling are examined in the embryonic brain and in the post-natal retina, a CNS extension that serves as a well-established complementary system for studying CNS angiogenesis (Milde et al., 2013). The findings show that Fbln1 is dispensable for early brain angiogenic patterning yet becomes essential for post sprouting vascular remodeling beginning at mid-gestation. In both the brain and retina, loss of Fbln1 disrupts venous derived microvascular compartments, producing dilated, tortuous capillaries that give rise to glomeruloid vascular lesions and intracerebral and intraretinal hemorrhages. Together, these results identify Fbln1 as a critical ECM regulator of endothelial behavior and vascular stability during CNS angiogenesis.

## 2. Materials and methods

### 2.1. Fbln1 floxed line

The targeting vector used to generate the conditional Fbln1-deficient mouse line (Fbln1 fl/fl) has been described previously (Okumura et al., 2024). All mouse experiments were conducted in accordance with guidelines from the Augusta University Institutional Animal Care and Use Committee and under an approved AU-IACUC animal protocol.

### 2.2. Genotyping of Fbln1 fl/fl mice

Genomic DNA was genotyped by polymerase chain reaction (PCR) using the following primers: forward common primer (5′-CCTTGCTCCCACCCCGGACATC-3′), wild-type-specific reverse primer (5′-CCAGAGAACCCCTGCTCTGCC-3′), and mutant-specific reverse primer (5′-CAGAGCCTCAGAAGAACTGATGGCGAGC-3′). PCR amplification generated a 528 bp product for the wild-type Fbln1 allele and a 628 bp product for the floxed Fbln1 allele. PCR was performed for 29 cycles consisting of denaturation at 95 C for 30 s, annealing at 55 C for 30 s, and extension at 72 C for 1 min.

### 2.3. Germline deletion of Fbln1 in mice

To delete the floxed exon 3, and generate Fbln1 ΔEx3/ΔEx3 mice (designed as Fbln1ko), female Fbln1 fl/fl mice were crossed with male 129S/Sv-Tg(Prm-cre)58Og/J mice (Jackson Laboratory, strain #003328), as previously described by O’Gorman et al. (O’Gorman et al., 1997). Offspring were screened for Cre-mediated recombination by PCR using the primers 5′-TGGATGAGAGCCATATTTGACATCCTTCCG-3′ and 5′-GAGGCCCTAGTGTGGGTCTGGTGTGAGC-3′. The wild-type Fbln1 allele produced an approximately 1026-bp amplicon, whereas the recombined allele lacking exon 3 produced an approximately 300-bp amplicon. PCR was performed for 29 cycles of 95 C for 30 s, 55 C for 30 s, and 72 C for 1 min 30 s.

### 2.4. Tamoxifen inducible deletion of floxed exon 3 in the Fbln1 gene

Tamoxifen-inducible B6.Cg-Tg(CAG-cre/Esr1*)5Amc/J mice (Jackson Laboratory, strain #004682) were crossed with Fbln1 fl/fl to generate Fbln1 fl/fl:CAG-Cre mice. These mice were crossed with Fbln1 ΔEx3/fl mice to produce Fbln1 ΔEx3/fl:CAG-Cre (designated iFbln1ko) for analysis. Tamoxifen (Millipore Sigma, #T5648) was prepared at 10 mg/ml by dissolving in 910 μl sunflower seed oil (Millipore Sigma, #S5007) with 90 μl of 100% ethanol, followed by sonication for 15 min. To induce Cre recombinase activity, postnatal pups received intragastric tamoxifen (50 μg at 1 μg/μl in sunflower seed oil), as previously described, on postnatal days P1, P3, and P6 for analysis at P8, or on P2, P4, and P5 for analysis at P12 (Pitulescu et al., 2010). Tamoxifen-treated animals lacking Cre served as controls, and both male and female mice were included in all experiments.

### 2.5. Immunohistochemistry of embryonic and postnatal tissues

Tissues, including E11.5 heads, embryonic brains, and postnatal retinas, were fixed in 4% paraformaldehyde in 1X PBS. Fixed samples were either vibratome-sectioned, processed through a sucrose/OCT gradient and frozen for cryosectioning, or dissected for whole-mount staining (E11.5 heads bisected along the midline; retinas cut into regions). Tissues or sections were permeabilized in 1X PBS containing 0.1% Triton X-100 for 1 h and blocked in 1% BSA in 1X PBS. Samples were incubated overnight at 4 C with biotinylated isolectin GS-B4 (Thermo Fisher Scientific, I21414) or primary antibodies, including rabbit anti-Fbln1 (2954) (Godyna et al., 1996), CD31 (Abcam, ab28364), type IV collagen (Abcam, ab6586), CD13 (MBL, m101-3), Ter119 (R&D Systems, MAB1125), or Endomucin (Santa Cruz, sc-65495). After washes in 1X PBS, bound lectin or primary antibodies were detected using Alexa Fluor-conjugated streptavidin or species-specific Alexa Fluor secondary antibodies or Goat anti-Rabbit IgG (H + L) secondary antibody, HRP (Thermo Fisher Scientific). Nuclei were counterstained with 1 μg/mL DAPI (Molecular Probes) for 10 min before final washes. Sections were mounted in ProLong Diamond Antifade Mountant (Thermo Fisher Scientific), and whole tissues were mounted in Cederlane RapiClear 1.52 (Fisher Scientific, RC152001) prior to imaging with a Leica Stellaris 5 confocal microscope.

### 2.6. PNVP, hindbrain and brain vessel analysis

Hindbrains were imaged by confocal microscopy. Radial vessel entry into the hindbrain (200 μm^2^ fields), vascular branch points in the PVP (200 μm^2^ fields), and PVP vessel diameters (200 μm^2^ fields) per embryo were quantified in 5-10 randomly selected fields per hindbrain. All analyses were performed using FIJI/ImageJ, with vascular diameters measured using the VasoMetrics macro (McDowell et al., 2021). PNVP vascular diameters obtained from confocal microscopy scans of E11.5 embryonic heads were quantified from three randomly selected 50 μm^2^ fields per embryo. Brain sections were imaged by confocal microscopy. Branch points and tip cells were quantified within 200 μm^2^ fields in at least five randomly selected regions per section along the angiogenic front. Filopodia per tip cell were quantified from at least 11 tip cells at the angiogenic front. All Analyses were performed using FIJI/ImageJ.

### 2.7. Pericyte and type IV collagen (Col IV) vascular coverage analysis

Brain sections were permeabilized with 0.1% Triton X-100 and stained with isolectin B4 and anti-CD13 or anti-Col IV. After secondary antibody labeling, sections were mounted and imaged by confocal microscopy. Pericyte and Col IV coverage was quantified in four randomly selected 200 μm^2^ fields per brain (N = 3 per genotype) using FIJI/ImageJ and AngioTool (Zudaire et al., 2011). Quantification of pericyte coverage and Col IV coverage on IB4-labeled vessels was expressed as the percentage of pericyte or Col IV area relative to IB4 area.

### 2.8. Measurement of retinal vascular outgrowth

Flat mounted retinas from postnatal day 8 (P8) mice were divided into regions and stained with isolectin B4. Superficial vascular outgrowth was quantified by measuring the radial distance from the center of the optic nerve head to the leading edge of the vascular plexus. Multiple measurements were taken within each region and averaged to generate a single outgrowth value per retina. To normalize for differences in retinal size, vascular outgrowth was expressed as a percentage: (distance from optic nerve head to vascular front ÷ distance from optic nerve head to retinal edge) x100. Three retinas from independent mice were analyzed, with each retina treated as one biological replicate.

### 2.9. Measurement of deep retinal vascular plexus

Flat-mounted retinas from postnatal day 12 (P12) mice were stained with isolectin B4 (IB4). The superficial and deep vascular plexuses were imaged by confocal microscopy, and maximum intensity projections were generated. Vessel density was quantified as the percentage of IB4-positive area within defined regions of interest in each retinal region, with measurements averaged to yield a single value per retina. Four retinas from independent mice were analyzed, with each retina considered a single biological replicate.

### 2.10. Western analysis

Protein concentration of tissue lysates was determined using the Bicinchoninic Acid (BCA) Protein Assay (Cat# 5000006, Bio-Rad, Hercules, CA). The tissue lysates were resolved by sodium dodecyl sulfatepolyacrylamide gel electrophoresis (SDS-PAGE), transferred to polyvinylidene difluoride (PVDF) membrane (Cat# IPVH00010, Immobilon-P; Millipore, Burlington, MA), and blocked in 5% milk in Tween-Tris buffered saline (TTBS) (10 mM Tris-HCl pH 7.6, 0.5% Tween 20, 150 mM NaCl). Western blot analysis was carried out using primary antibodies against Fbln1 (2954) and GAPDH (Cell Signaling Technology (14C10,Cat# 2118). After primary antibody incubation, membranes were washed in TTBS and subsequently incubated with species-specific horseradish peroxidase-conjugated secondary antibodies. Membranes were washed in TTBS, and specific signals were visualized using an enhanced chemiluminescence substrate (Cat# NEL122001EA, ECL-Plus; PerkinElmer, Waltham, MA) an a ChemiDoc Touch Imaging System (Biorad). Densitometry analysis was performed using FIJI/ImageJ and normalized to the loading control.

### 2.11. Statistics

Statistical analyses were performed using GraphPad Prism 6 software. Differences between groups were assessed using an unpaired two-sample *t*-test. Data are presented as mean ± standard error of the mean (SEM). A *P* value < 0.05 was considered statistically significant.

## 3. Results

### 3.1. Characterization of the floxed Fbln1 mouse model

Loss of Fbln1 results in a pronounced bleeding phenotype, however, the underlying reason remains unclear (Cooley et al., 2008; Kostka et al., 2001). To investigate the onset and cause of Fbln1-associated hemorrhage, a floxed Fbln1 mouse line targeting exon 3 (designated Fbln1 fl/fl) (Okumura et al., 2024) was used to generate both germline and tamoxifen-inducible Fbln1-deficient models. Because Fbln1 null mice die at birth, the use of an inducible Cre system enables investigation of the role of Fbln1 in vascular development, with particular emphasis on embryonic CNS and postnatal retinal angiogenesis.

To generate a germline Fbln1-deficient line, Fbln1 fl/fl mice were first crossed with Protamine-Cre mice to produce Fbln1 +/ΔEx3 heterozygous animals. These heterozygotes were then intercrossed to generate Fbln1 ΔEx3/ΔEx3 null embryos (designated Fbln1ko) for phenotypic analysis (Fig. 1A). Immunostaining and Western blot analyses of tissues from control and Fbln1ko embryos confirmed loss of Fbln1 following exon 3 deletion (Fig. 1B and C,D). Intercrosses of heterozygous Fbln1 mice failed to produce viable adult homozygous mutants (Fbln1ko), resulting in a non-Mendelian genotype distribution (Table 1), consistent with prior reports using the previously described gene-trap Fbln1 deletion model (Fbln1Gt^(XST011)Byg^) (Cooley et al., 2008) and the non-conditional exon 1 deletion line Fbln1^tm1Tmpl^ (Kostka et al., 2001).

To define the developmental timing of the vascular defect associated with Fbln1 loss, Fbln1ko embryos were examined from E11.5 to E17.5. At E11.5, Fbln1ko embryos were morphologically indistinguishable from controls (Fig. 2A). By E12.5, approximately 20% of Fbln1ko embryos exhibited intracerebral hemorrhage (Table 1), and by E13.5, this phenotype was fully penetrant (Fig. 2B–Table 1). At E13.5, hemorrhages were also observed in the skin (Fig. 2). Both the severity and anatomical distribution of hemorrhage increased progressively with developmental stage (data not shown). This temporal progression and tissue specificity are consistent with previously reported phenotypes in the Fbln1Gt^(XST011)Byg^ (Cooley et al., 2008) and Fbln1^tm1Tmpl^ deficient mouse models (Kostka et al., 2001).

### 3.2. Fbln1-deficient vessels exhibit normal early embryonic CNS vascular development

To determine whether loss of Fbln1 affects the formation of large superficial vessels or the perineural vascular plexus (PNVP) that surrounds the developing CNS prior to hemorrhage, CD31 and endomucin immunostaining was performed on cranial regions from E11.5 control and Fbln1ko embryos. CD31 labeling revealed comparable large-caliber surface vessels and a dense PNVP capillary network encircling the neuroepithelium in both genotypes, with no overt differences in vascular organization (Fig. 3A). Similarly, endomucin immunostaining, which preferentially labels venous and capillary endothelium, revealed a similar pattern of head veins and PNVP capillary networks in control and Fbln1ko embryos (Fig. 3B) (Park-Windhol et al., 2023). In addition, analysis of PNVP vessels demonstrated similar vascular diameters between the genotypes (Fig. 3C) indicating that loss of Fbln1 does not disrupt PNVP architecture prior to hemorrhage.

To assess whether Fbln1 is required for angiogenic sprouting from the PNVP into the neuroepithelium, isolated E11.5 hindbrains were stained with isolectin B4 (IB4) to visualize endothelial extensions from the PNVP. Endothelial sprouts were observed to enter the outer surface of the hindbrain and evenly distribute, with no detectable morphological differences between genotypes (Fig. 4A). These sprouts traversed the hindbrain and branched to establish the periventricular vascular plexus (PVP) (Fig. 4B). Quantitative analyses confirmed that loss of Fbln1 did not alter the number of sprouts entering the outer hindbrain surface, nor did it affect vessel diameter or branching complexity of vessels within the PVP (Fig. 4C). Immunofluorescence analysis showed that Fbln1 localized to the vascular BM of the PNVP, as well as to invading sprouts and endothelial stalk cells during PVP formation, but was largely absent from endothelial tip cell filopodia (Fig. 4D). These findings indicate that Fbln1 is dispensable during early stages of CNS angiogenesis, including sprout initiation and ingression, and instead support a role for Fbln1 in promoting microvascular stabilization at later developmental stages.

### 3.3. Fbln1 deficiency leads to the formation of glomeruloid vessels in the CNS during mid-gestation

Beginning at approximately E15.5, Fbln1ko embryos exhibited dilated, tortuous capillaries that gave rise to glomeruloid vascular lesions, features absent in control brains. These abnormalities became more pronounced at later embryonic stages and were preferentially localized to the angiogenic front in the periventricular regions (Fig. 5A). These lesions showed a relative reduction in outwardly extending angiogenic filopodia (Fig. 5B). However, quantification of tip cells, filopodia per tip cell and vascular branch points at the angiogenic front was not significantly different between genotypes, indicating that tip cell morphology and filopodial initiation were largely preserved, allowing continued cerebral angiogenic front progression (Fig. 5C). Similar glomeruloid structures were also observed in the meninges of Fbln1-null embryos (Fig. 6A).

Immunofluorescence analysis of cerebral sections using IB4 and anti-Ter119 to label erythrocytes demonstrated that many of these glomeruloid structures contained patent, erythrocyte-filled lumens. Evidence of vascular leakage was also observed, indicated by extravasated Ter119^+^ cells in the surrounding parenchyma (Fig. 6B).

### 3.4. Glomeruloid structures in Fbln1ko brains exhibit reduced Col IV deposition

The presence of glomeruloid vascular structures in Fbln1ko embryos suggested that compromised vascular BM integrity might contribute to lesion expansion into the brain parenchyma. To investigate this possibility, immunofluorescence staining was performed on cerebral sections using type IV collagen (Col IV) antibodies to label the BM and IB4 to delineate endothelial lumens. In both genotypes, IB4 and Col IV displayed normal apical and basal localization, respectively, and control tissue exhibited uniform Col IV coverage (Fig. 7A). In contrast, glomeruloid lesions in Fbln1ko embryos displayed focal reductions in Col IV deposition (Fig. 7A, *white arrowheads*). Despite this localized reduction surrounding glomeruloid lesions, quantitative assessment of Col IV (Fig. 7B), within the BM of cerebral vessels revealed no significant differences between genotypes. These findings indicate that Fbln1 is not required for overall BM integrity, consistent with prior ultrastructural analyses of Fbln1^tm1Tmpl^ mice, which reported no major BM defects (Kostka et al., 2001).

### 3.5. Fbln1 deficiency does not alter pericyte investment of cerebral vessels

During CNS development, pericytes contribute to the vascular BM by depositing ECM components and engaging in bidirectional signaling with ECs, interactions known to support endothelial maturation, quiescence, and barrier integrity (Payne et al., 2020; Armulik et al., 2010). These functions raised the possibility that the hemorrhages observed in Fbln1ko embryos might result from defects in pericyte investment. To assess this, immunofluorescence analysis was performed on E16.5 cerebral sections stained with IB4 to label ECs and CD13 as a pericyte marker (Smyth et al., 2018). In control brains, CD13^+^ pericytes were closely associated with ECs, providing near-continuous vascular coverage and extending along endothelial stalk cells at newly formed branches (Fig. 8).

In Fbln1ko embryos, CD13^+^ pericytes were distributed throughout the cerebral vasculature (Fig. 8A), although the glomeruloid lesions lacked clear pericytes coverage (Fig. 8B, white arrowheads). In addition, CD13 immunoreactivity was also detected on blood cells within glomeruloid lesions, consistent with its known expression in myeloid lineage cells (Fig. 8B, *yellow arrowhead*) (Winnicka et al., 2010). Despite these localized alterations in pericyte-endothelial interactions, quantitative analysis revealed no significant difference in overall pericyte coverage between genotypes (Fig. 8C), indicating that a global defect in pericyte investment is unlikely to underlie the hemorrhagic phenotype in Fbln1ko embryos. Consistent with previous studies, reduced pericyte-endothelial association is more commonly associated with impaired vascular barrier maturation and leakage of small molecular weight molecules rather than overt hemorrhage (Armulik et al., 2010; Daneman et al., 2010; Arnold et al., 2014; Hellström et al., 2001).

### 3.6. Deletion of Fbln1 affects vein and capillary development

Immunostaining of Fbln1ko brains with IB4 and CD31 suggested that the glomeruloid vascular lesions primarily arose from capillaries and vessels with venous-like morphology. In control embryos, CD31 exhibited strong, continuous localization along endothelial cell-cell borders, whereas glomeruloid lesions in Fbln1ko embryos displayed markedly reduced or absent CD31 immunoreactivity, with adjacent morphologically normal vessels retaining continuous endothelial labeling (Fig. 9). The reduced and discontinuous CD31 staining within the lesions is consistent with altered endothelial organization. This phenotype in Fbln1ko brains resembles human vascular malformations that exhibit reduced CD31 and VE-cadherin expression together with aberrant venous specification associated with the ectopic expression of arterial markers (Schonning et al., 2021).

Because arteries and veins cannot be reliably distinguished in the embryonic brain based on morphology or available immunolabeling methods, the postnatal retina was examined to further define the affected vascular beds. The retinal vasculature contains anatomically distinct arteries, veins, and capillaries, enabling vessel-specific analysis. In addition retinal angiogenesis begins after birth (approximately postnatal day 1 in mice), providing a well-defined and experimentally accessible model for investigating the role of Fbln1 in vascular development (Pitulescu et al., 2010).

To bypass embryonic lethality of constitutive Fbln1 deficiency, Fbln1ΔEx3/fl mice were crossed with a tamoxifen-inducible ubiquitous Cre line (B6.Cg-Tg(CAG-cre/Esr1*)5Amc/J) to generate Fbln1 ΔEx3/fl: CAG-Cre mice (designated iFbln1ko) (Fig. 10A and B). Pups received tamoxifen on postnatal day (P) P1, P3, and P6, and retinas from induced mutants and Cre-negative controls were analyzed at P8. iFbln1ko retinas at P8 appeared grossly normal in overall morphology but consistently exhibited focal hemorrhages (Fig. 10C). IB4 staining revealed a significant reduction in radial expansion of the superficial vascular plexus (Fig. 10D and **E *n*** = ***3 per genotype, P*** = ***0.0293*)** and the presence of tortuous capillaries with glomeruloid vascular lesions (Fig. 11A). Ter119 immunolabeling confirmed extravasated erythrocytes within the retina. All of these phenotypes were absent in control retinas. Many glomeruloid structures also contained erythrocyte filled lumens (Fig. 11A), resembling the vascular abnormalities observed in Fbln1ko embryonic brains (Fig. 6B), indicating a conserved requirement for Fbln1 in maintaining vascular integrity.

During normal development of the retina, superficial veins and adjacent capillaries initiate vertical sprouting around P7 to generate the deep vascular plexus, which becomes largely established by P10-P12, followed by formation of the intermediate plexus (Milde et al., 2013). To assess the impact of Fbln1 loss on deep plexus vascularization, pups were administered tamoxifen at P2, P4, and P5, and retinas from induced mutants and Cre negative controls were analyzed at P12. Immunofluorescence confirmed that at P12, Fbln1 protein localized to both superficial vessels and the deep vascular plexus (Fig. 11B), and that tamoxifen induced Cre activity effectively reduced Fbln1 expression within vertical sprouts in iFbln1 ko retinas (Fig. 11B).

Confocal imaging of IB4 stained P12 retinas showed that, in controls, vertical sprouts originated from the reverse side of superficial veins and adjacent capillaries (Fig. 12,***yellow arrows*),** whereas superficial arteries remained non-sprouting, with surrounding capillaries only showing vertical sprouts (Fig. 12, **white asterisks)**, consistent with prior reports (Milde et al., 2013; Lee et al., 2021). In iFbln1ko retinas, prominent glomeruloid lesions were observed not only within the surface view of the superficial plexus but also along descending sprouts from the reverse side of superficial veins and capillaries (Fig. 12B **yellow arrowheads)**, while superficial arteries remained quiescent, with surrounding capillaries only showing glomeruloid lesions from vertical sprouts (Fig. 12 A, ***yellow asterisks*).** These glomeruloid abnormalities were associated with a significant reduction in deep plexus density (Fig. 13A and B*;* ***n*** = ***4 per genotype, P*** = ***0.0091*)**, ranging from localized vessel loss to hypoplastic networks. Cross sectional analyses further demonstrated that many vertical sprouts terminated in glomeruloid lesions, indicating that Fbln1 is required for proper vertical sprouting and deep retinal vascular network formation (Fig. 13C).

## 4. Discussion

Fbln1, the prototypical member of the fibulin family, is essential for cardiac development (Cooley et al., 2008, 2012; Ito et al., 2020; Harikrishnan et al., 2015; Argraves et al., 2003) and promotes intimal hyperplasia following vascular injury (Okumura et al., 2024). However, its role in microvascular development has remained unclear. Using both germline and inducible models of Fbln1 deletion, we define a stage-specific requirement for Fbln1 during CNS and postnatal retinal angiogenesis.

In germline Fbln1-deficient embryos, cranial hemorrhage emerged at E12.5 with incomplete penetrance and became fully penetrant by E13.5 and worsened with gestation age. Hemorrhage during this developmental window in other mouse models is often associated with defects in superficial cranial vessels and the PNVP (Cullen et al., 2011; Pitter et al., 2018). However, examination of Fbln1 null embryos at E11.5, prior to the onset of hemorrhage, revealed that superficial cranial vessels, the PNVP, and hindbrain vascular development, including sprouting from the PNVP to form the PVP, were morphologically indistinguishable from controls. These findings indicate that Fbln1 is dispensable for early arterial-venous patterning and initial CNS angiogenesis.

Although defects in the initial establishment of the blood brain barrier (BBB), which begins around E12.5 and becomes functional by E15.5, have been proposed as a potential cause of intracerebral hemorrhage, several lines of evidence argue against a primary defect in early barrier specification in Fbln1ko embryos (Cullen et al., 2011; Daneman et al., 2009; Sadanandan et al., 2024; Zhou and Nathans, 2014; Zhou et al., 2014). Pericyte investment, a critical early step in BBB induction, is preserved, and angiogenic sprouting and vascular patterning appear intact prior to hemorrhage, indicating that initial barrier programs are properly established (Daneman et al., 2010). Moreover, Itgb8-deficient mice hemorrhage during this same window due to abnormal sprouting despite an intact endothelial barrier (Arnold et al., 2014) where as Pdgfb hypomorphs exhibit reduced pericyte vascular coverage and BBB dysfunction without overt hemorrhage (Armulik et al., 2010; Daneman et al., 2010; Arnold et al., 2014). Together, these findings suggest Fbln1 loss does not impair early BBB induction.

Instead, hemorrhage in Fbln1 deficient brains coincides with aberrant vascular remodeling and impaired endothelial barrier maturation in response to blood flow, processes that rely on ECM-dependent stabilization of endothelial junctions and lumen formation (Kim et al., 2017; Yamamoto et al., 2015; Nakajima et al., 2017). Consistent with this, Fbln1 deficient brains and retinas develop glomeruloid vascular structures, thought to arise when angiogenic sprouts fail to remodel into stable, perfused vessels (Cullen et al., 2011; Zhou et al., 2014). These lesions in Fbln1 nulls exhibit heterogeneous lumen organization, with erythrocyte filled vessels adjacent to endothelial lined but non perfused channels, indicating incomplete or unstable lumen formation. Like glomeruloid lesions in other mouse models, these structures contain few filopodia, suggesting they are not active sprouting sites (Cullen et al., 2011; Kim et al., 2017). Multiple studies indicate that such lesions reflect defective coordinated EC migration, a process essential for vascular expansion under the hypoxic conditions of the developing brain and retina (Kim et al., 2017; Dubrac et al., 2016; Sakabe et al., 2017). Despite these glomeruloid abnormalities, tip cell formation and filopodia number per tip cell remain unchanged in Fbln1 deficient brains, suggesting that Fbln1 regulates endothelial migration rather than tip cell specification.

Glomeruloid lesions in Fbln1-deficient brains also exhibit reduced incorporation of the BM component type IV collagen. Abnormal ECM incorporation is also characteristic of Cerebral Cavernous Malformations, where increased ECM remodeling facilitates lesions expansion into the surrounding brain parenchyma (Hong et al., 2020; Schwefel et al., 2020; Schneider et al., 2011). Interestingly, these VEGF-driven malformations also display variable size with reduced tip cell formation and are also associated with defective EC migration (Castro et al., 2019; Laviña et al., 2018). Consistent with enhanced BM remodeling, VEGF signaling at angiogenic fronts has been shown to coincide with proteolysis of type IV collagen and incomplete basement membrane deposition during angiogenesis, suggesting that abnormal sprouting activity may disrupts coordinated BM assembly and impairs vascular stabilization (Spuul et al., 2016). Collectively, these findings suggest that Fbln1 primarily supports vascular remodeling, particularly vessel stabilization and barrier maturation necessary to prevent hemorrhage.

In Fbln1-deficient brain and retina, glomeruloid lesions are enriched along veins and capillaries, near the angiogenic front. This is notable because venous-derived ECs contribute extensively to CNS and retinal vascular networks (Lee et al., 2021). During retinal development, vertical sprouts emerge from superficial veins and adjacent capillaries, invade the inner retina, and branch laterally at the interface between the inner nuclear layer and the outer plexiform layer (INL-OPL) to establish the deep retinal plexus. In Fbln1 deficient retinas, ECs instead form aberrant aggregates (e.g. glomeruloid structures) that impede vertical invasion and reduce branching complexity, resulting in persistent avascular regions within the deep vascular plexus. These findings indicate that Fbln1 is a key regulator of venous-derived EC behavior likely by modulating endothelial cell-ECM interactions.

Mechanistically, Fbln1 may influence angiogenesis through its binding to the heparin-binding fragment of Fn containing type III repeats 13 -14. This region is distinct from the integrin-binding site (FnIII 9-10) and does not interfere with α5β1 integrin binding, the primary Fn receptor in ECs and a major driver of angiogenic growth (Twal et al., 2001; Yamamoto et al., 2015; Schumacher et al., 2021; Balbona et al., 1992; Li et al., 2012). Although Fbln1 can inhibit Fn-mediated migration in some cell types (Lambert et al., 2020; Twal et al., 2001), our findings indicate that in the CNS, Fbln1 is required for EC migration, highlighting a context-dependent role for this ECM protein in angiogenesis. It is plausible that Fbln1 supports persistent, directed migration under hypoxic conditions, particularly within the inner retina, which experiences low oxygen tension due to the high metabolic demand of retinal neurons and ongoing vascular development (Rattner et al., 2019).

Interestingly, glomeruloid lesions and disrupted deep retinal vascularization occur in mouse models that perturb Norrin/Wnt/β catenin (Ye et al., 2009),TGFβ (Allinson et al., 2012; Zarkada et al., 2021), and integrin linked kinase (ILK) signaling (Park et al., 2019), pathways implicated in Familial Exudative Vitreoretinopathy pathogenesis (Wang et al., 2025). Because Fbln1 can associate with Fn without blocking α5β1 binding, it is notable that EC-specific ILK loss produces a retinal phenotype similar to Fbln1 deficiency, suggesting that ILK may function downstream of Fbln1. Surprisingly, EC-specific integrin β1 loss, despite disrupting deep plexus formation and binding ILK, does not produce glomeruloid lesions (Yamamoto et al., 2015; Hannigan et al., 1996). This phenotypic distinction suggests that Fbln1 may integrate multiple signaling inputs, potentially including growth factor receptors and integrin-mediated cues, to coordinate endothelial behavior and promote vessel stabilization.

In conclusion, our findings support a model in which Fbln1 acts as a context-dependent modulator of endothelial migration and microvascular network maturation, bridging ECM and signaling cues to ensure proper CNS vascular development. Future studies will determine whether Fbln1 modulates ILK or other integrin mediated pathways to promote EC migration and barrier maturation in the CNS.

## Figures and Tables

**Fig. 1.**
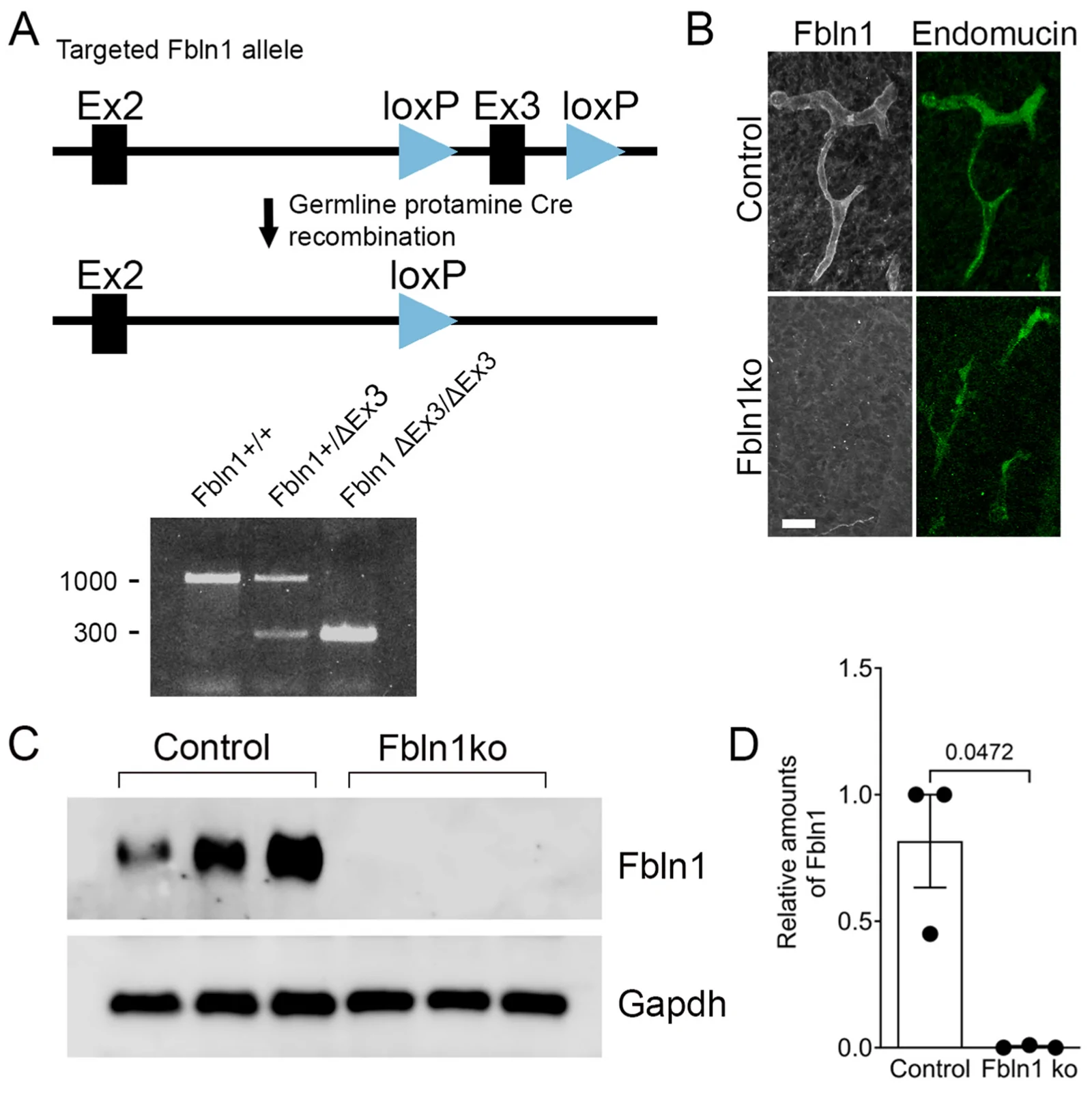
Deletion of Exon 3 in the Fbln1 allele abolishes Fbln1 expression. (A) Schematic of the Fbln1 allele showing loxP sites flanking exon 3 (top) and germline deletion of exon 3 following the crossing Fbln1 floxed mice with a protamine Cre line (bottom). PCR genotyping of DNA isolated from wild type (+/+), heterozygous (+/ΔEx3), and homozygous (ΔEx3/ΔEx3, designated as Fbln1ko) embryos. (B) Immunofluorescence of embryonic brain sections stained for endomucin and Fbln1 (antibody 2954). In controls, Fbln1 is deposited in the extracellular matrix surrounding brain capillaries, whereas it is absent in Fbln1ko sections. Scale equals 25 μm (C) Immunoblot of embryonic tissue lysates probed with Fbln1 (antibody 2954) and GAPDH.Robust Fbln1 expression is detected in controls, whereas Fbln1 knockout lysates show no detectable protein. (D) Densitometric quantification of Fbln1 normalized to GAPDH confirms loss of expression in Fbln1 knockout tissue. N = 3 embryos per genotype. Black circles represent individual data points. Values are presented as mean ± SEM, and the *P* value is indicated above the bracket.

**Fig. 2.**
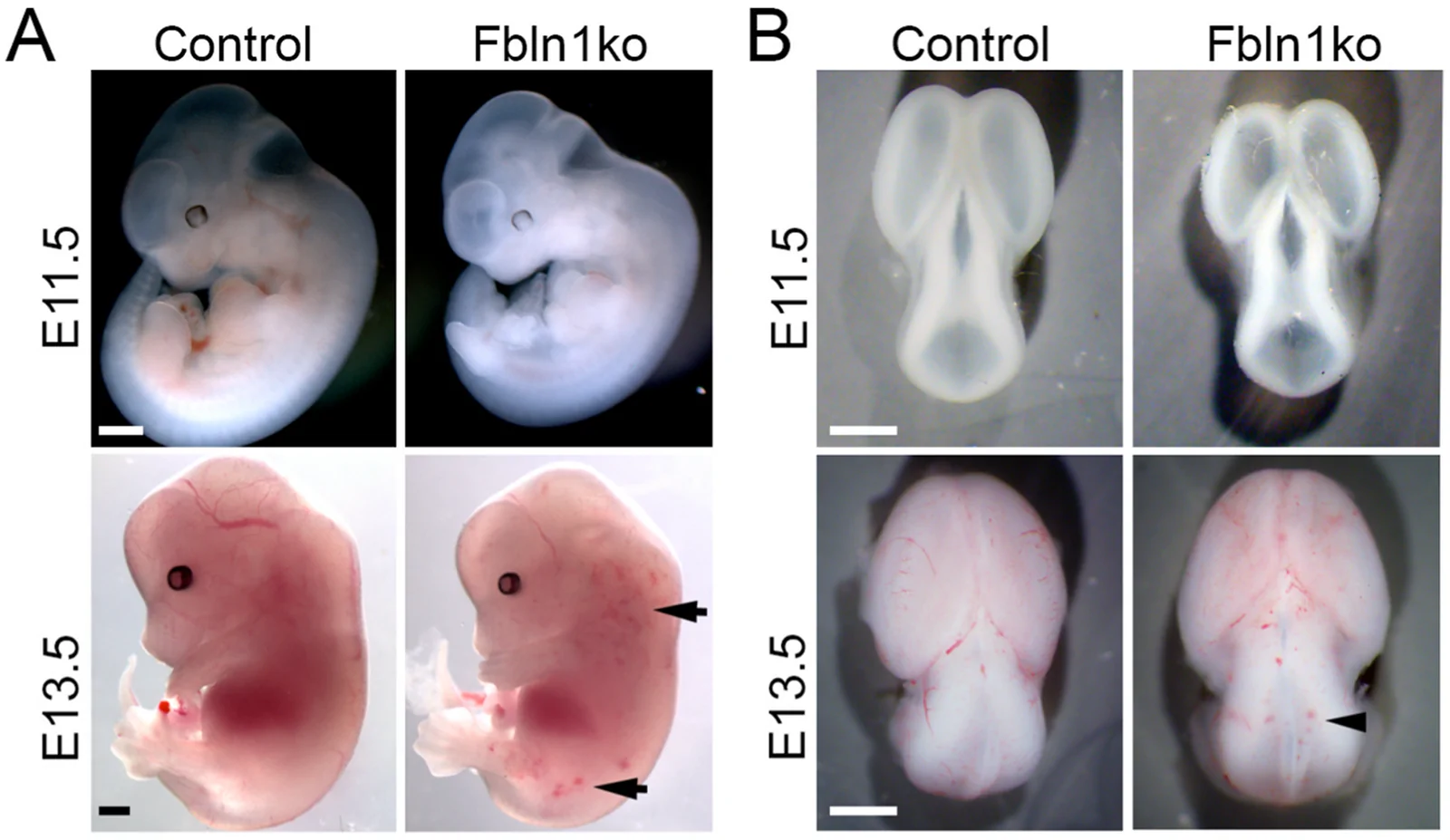
Deletion of Fbln1 causes cerebral hemorrhage. (A) Control and Fbln1ko embryos show comparable gross morphology at E11.5. Scale bar,1 mm. By E13.5, Fbln1ko embryos exhibit overt hemorrhages (black arrows) absent in controls. Scale bar,1 mm. (B) Control and Fbln1ko brains appear morphologically similar at E11.5. Scale bar, 500 μm. By E13.5, Fbln1 ko brains display cerebral hemorrhage (black arrowhead) not observed in controls. Scale bar,500 μm.

**Fig. 3.**
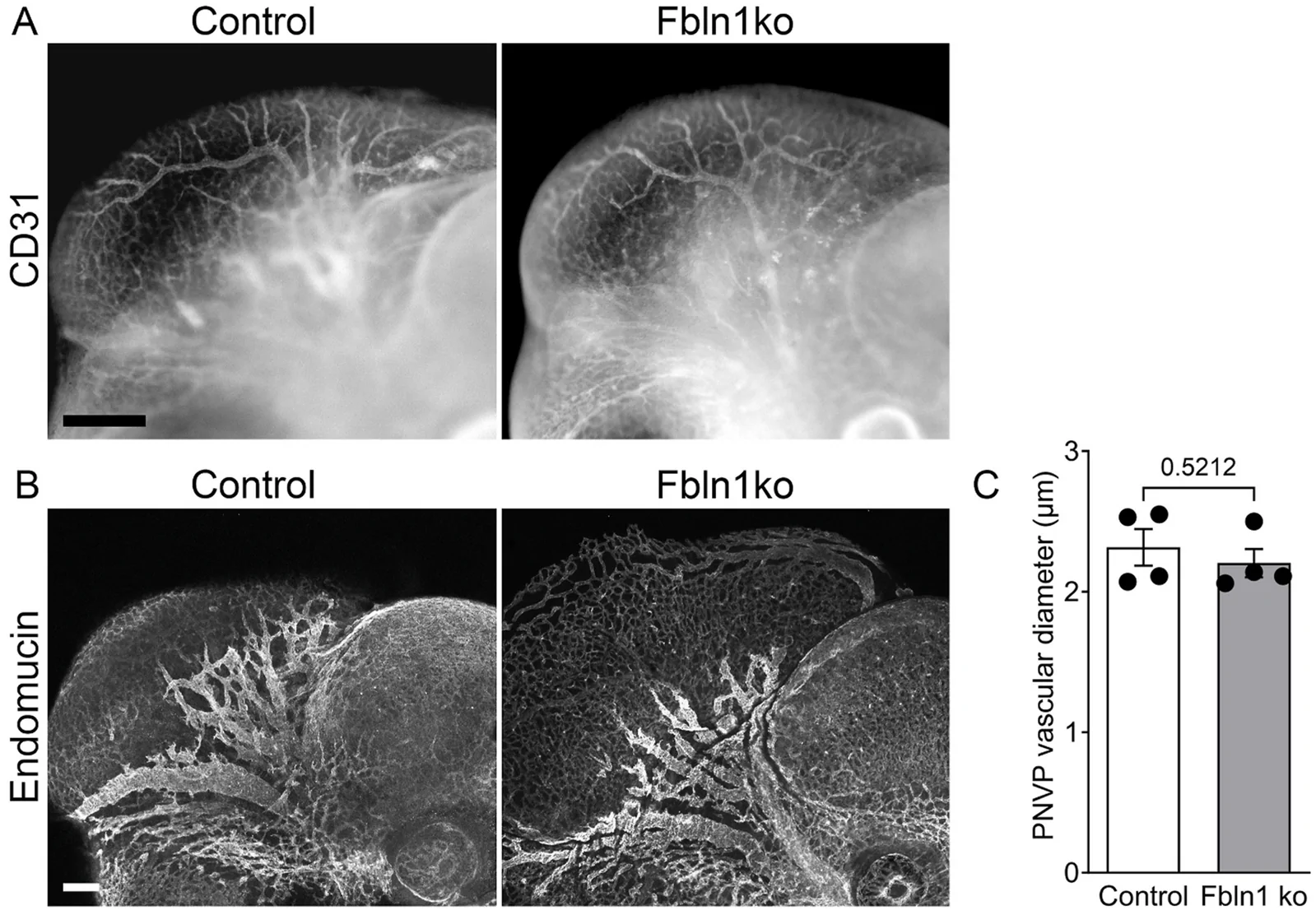
Loss of Fbln1 does not affect early superficial vessel formation or PNVP capillary development. Immunostaining of E11.5 control and Fbln1ko cranial regions with (A) CD31 and (B) endomucin shows that, in both genotypes, large-caliber surface vessels and the perineural vascular plexus (PNVP) capillary network encircling the neuroepithelium exhibit no overt differences in vascular organization. (C) Quantification of PNVP vessel diameter. N = 4 embryos per genotype. Black circles represent individual data points. Values are presented as mean ± SEM, and the *P* value is indicated above the bracket. Scale bars, 500 μm (A) and 200 μm (B).

**Fig. 4.**
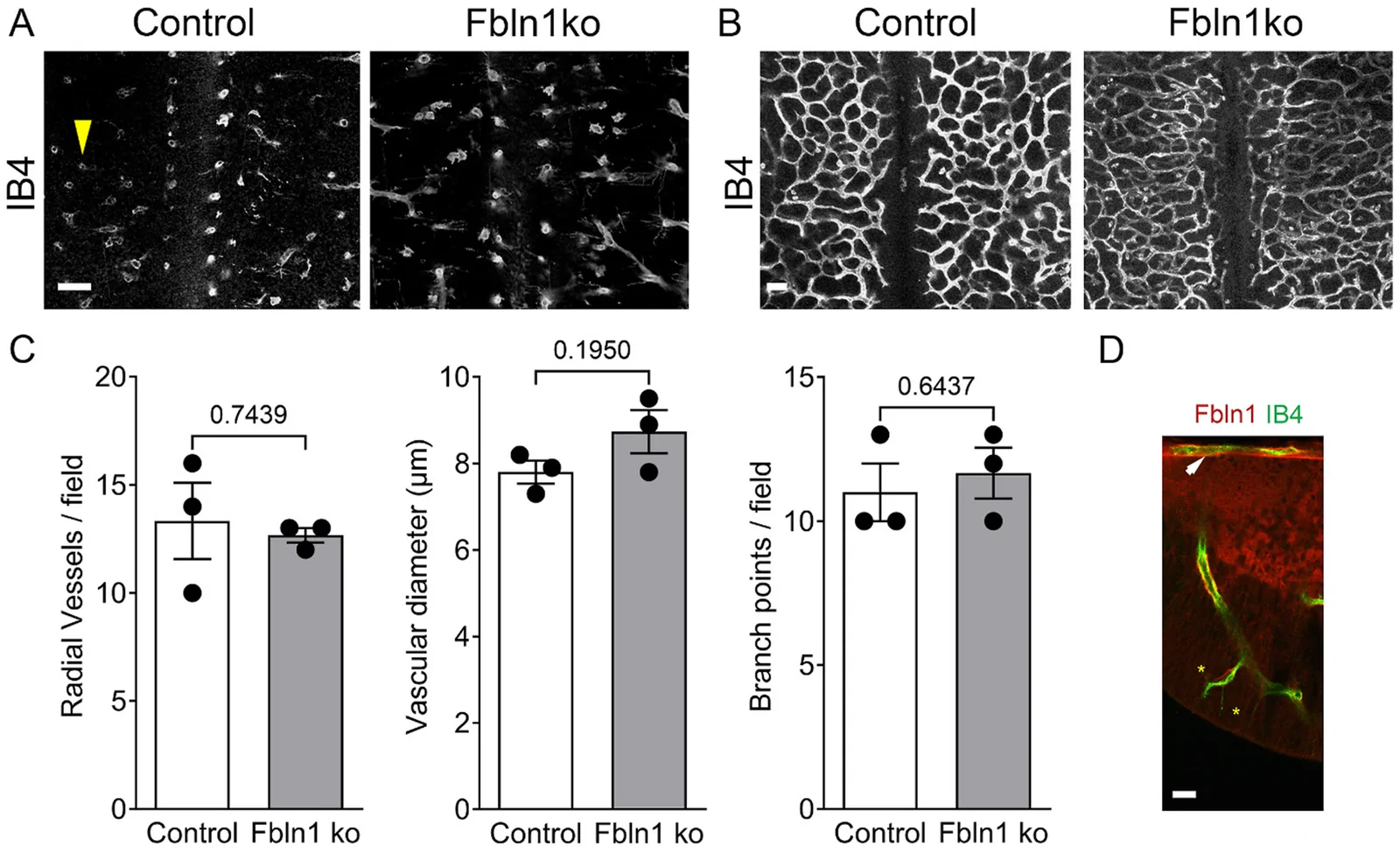
Analysis of E11.5 hindbrain vasculature in control and Fbln1ko embryos using isolectin B4. Z-stack projections of E11.5 hindbrains (A) show sites where PNVP radial sprouts enter the outer surface of the hindbrain (yellow arrowhead), (B) show radial vessels traversing the hindbrain and branching to form the PVP along the ventricular surface. Scale bars in A and B, 50 μm. (C) Graphs quantifying the number of radial vessels entering the hindbrain surface, vessel diameter and the number of branch points along the ventricular surface. N = 3 hindbrains per genotype. Black circles represent individual data points. Values represent mean ± SEM and the *P* value is indicated above the bracket.(D) Fbln1 immunostaining of an E11.5 hindbrain cross-section showing colocalization of Fbln1 (red) with isolectin B4-labeled vessels (green) in the hindbrain region and the PNVP vasculature (white arrow). The yellow asterisks show filopodia extending from tip cell that lack Fbln1 immunostaining. Scale bar, 25 μm. (For interpretation of the references to colour in this figure legend, the reader is referred to the Web version of this article.)

**Fig. 5.**
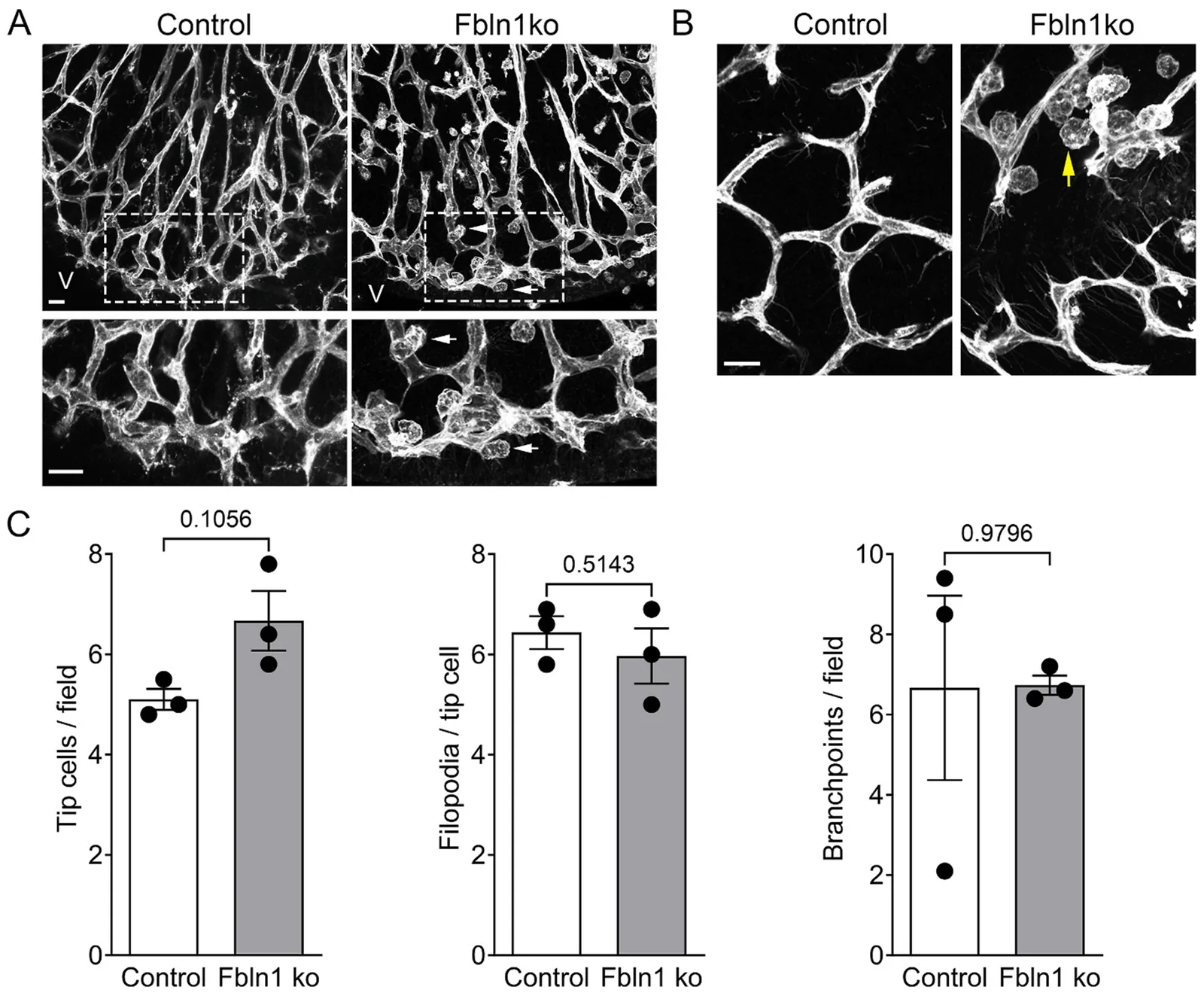
Fbln1ko brains display tortuous capillaries that give rise to glomeruloid vascular lesions. Z-stack projections of (A) cerebral sections from E17.5 control and Fbln1ko embryos stained with isolectin B4 (IB4). Tortuous capillaries with vascular lesions are evident in the paraventricular region of Fbln1ko brains (white arrows). Boxed regions are shown at higher magnification, illustrating normal vascular architecture in controls and glomeruloid malformations in Fbln1ko tissue (lower panels). (B) Vascular filopodia extending from control vessels and the reduced number of organized sprouts emerging from Fbln1ko vascular malformations (yellow arrow). (C) Quantification of tip cells, filopodia per tip cell, and vascular branch points at the angiogenic front in E17.5 control and Fbln1ko brains. N = 3 brains per genotype; Black circles represent individual data points. Values are presented as mean ± SEM, and the *P* value is indicated above the bracket. Scale bars, 25 μm (A, B). V, ventricle. (For interpretation of the references to colour in this figure legend, the reader is referred to the Web version of this article.)

**Fig. 6.**
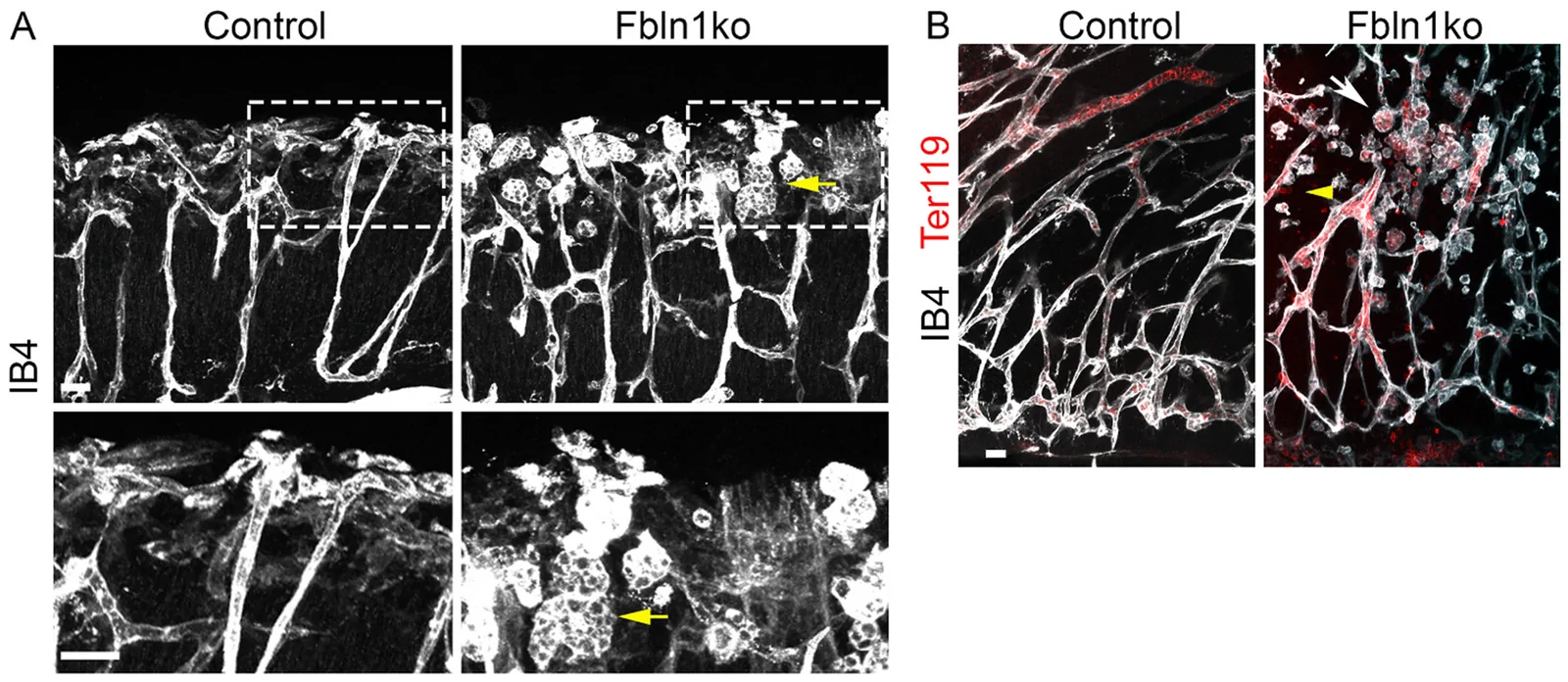
Fbln1 deficiency leads to glomeruloid vascular lesions in the meninges and the presence of erythrocytes within the lumens of cerebral vascular lesions. (A) Z-stack projections of immunofluorescence images from E17.5 brain sections stained with isolectin B4 (IB4) reveal glomeruloid vascular lesions (yellow arrow) in the meninges of Fbln1ko embryos. Higher magnification of the boxed regions shows normal vasculature in controls and vascular malformations in Fbln1ko brains (yellow arrow, lower panel). Scale bars (top and bottom panels), 25 μm. (B) Z-stack projections of immunofluorescence images from E17.5 brain sections stained with IB4 to label vessels and Ter119 to label erythrocytes. Fbln1ko brains exhibit vascular lesions with lumens containing blood cells (white arrow). The presence of vascular leakage was also observed, indicated by extravasated Ter119+ cells in the surrounding parenchyma (yellow arrowhead). Scale bar,25 μm. (For interpretation of the references to colour in this figure legend, the reader is referred to the Web version of this article.)

**Fig. 7.**
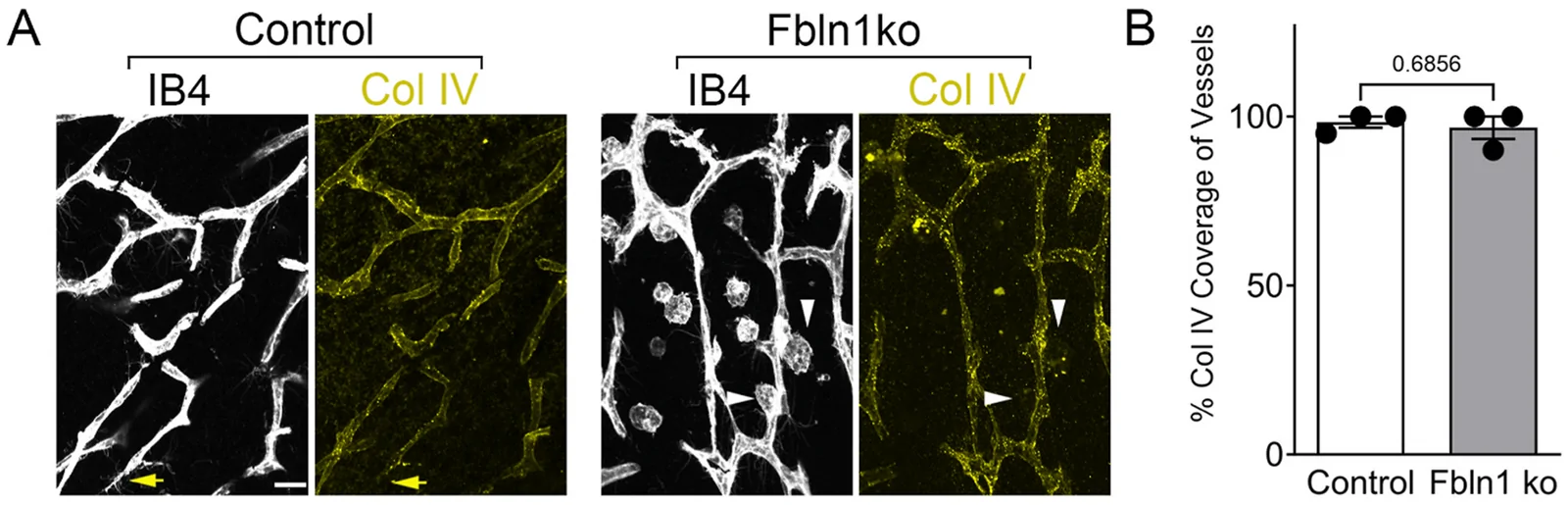
Glomeruloid lesions in Fbln1ko brains show reduced Col IV deposition. (A) Z-stack projections of brain sections from E16.5 control and Fbln1ko embryos stained with anti-Col IV and isolectin B4 (IB4). In controls, Col IV is deposited in the BM of vessels and tip cells (yellow arrow) but is absent around filopodia. In Fbln1ko brains, glomeruloid lesions (white arrowheads) show reduced Col IV deposition in the BM. (B) Quantification of Col IV coverage on IB4-labeled vessels, expressed as the percentage of Col IV area relative to IB4 area. N = 3 embryos per genotype; Black circles represent individual data points. Values are presented as mean ± SEM, and the *P* value is indicated above the bracket. Scale bar, 25 μm. (For interpretation of the references to colour in this figure legend, the reader is referred to the Web version of this article.)

**Fig. 8.**
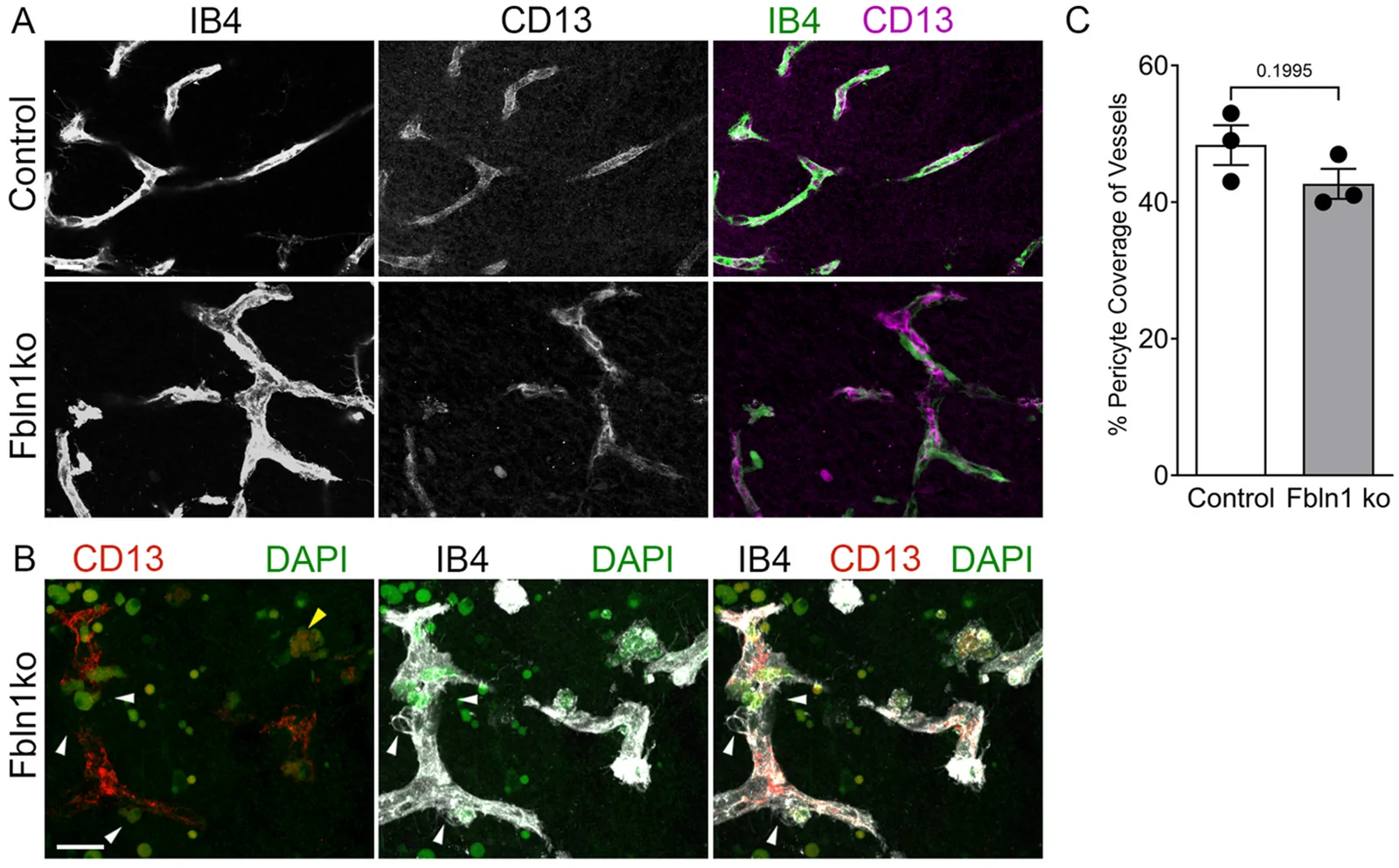
Fbln1 deficiency does not affect pericyte coverage of cerebral vessels. (A) Z stack projections of brain sections from E16.5 control and Fbln1ko embryos stained for CD13 (pericyte marker) and isolectin B4 (IB4). (B) Z-stack projection of Fbln1ko brain section stained for CD13, IB4 and DAPI to visualize nuclei. Note the presence of glomeruloid structures in the Fbln1ko brain that lack pericyte coverage (white arrowheads). The yellow arrowhead indicates CD13^+^ blood cells, consistent with the known expression of CD13 in myeloid lineage cells. (C) Quantification of pericyte coverage of IB4-labeled vessels, expressed as the percentage of CD13^+^ area relative to IB4 area. N = 3 embryos per genotype. Black circles represent individual data points. Values are presented as mean ± SEM, and the *P* value is indicated above the bracket. Scale bar, 25 μm (A,B). (For interpretation of the references to colour in this figure legend, the reader is referred to the Web version of this article.)

**Fig. 9.**
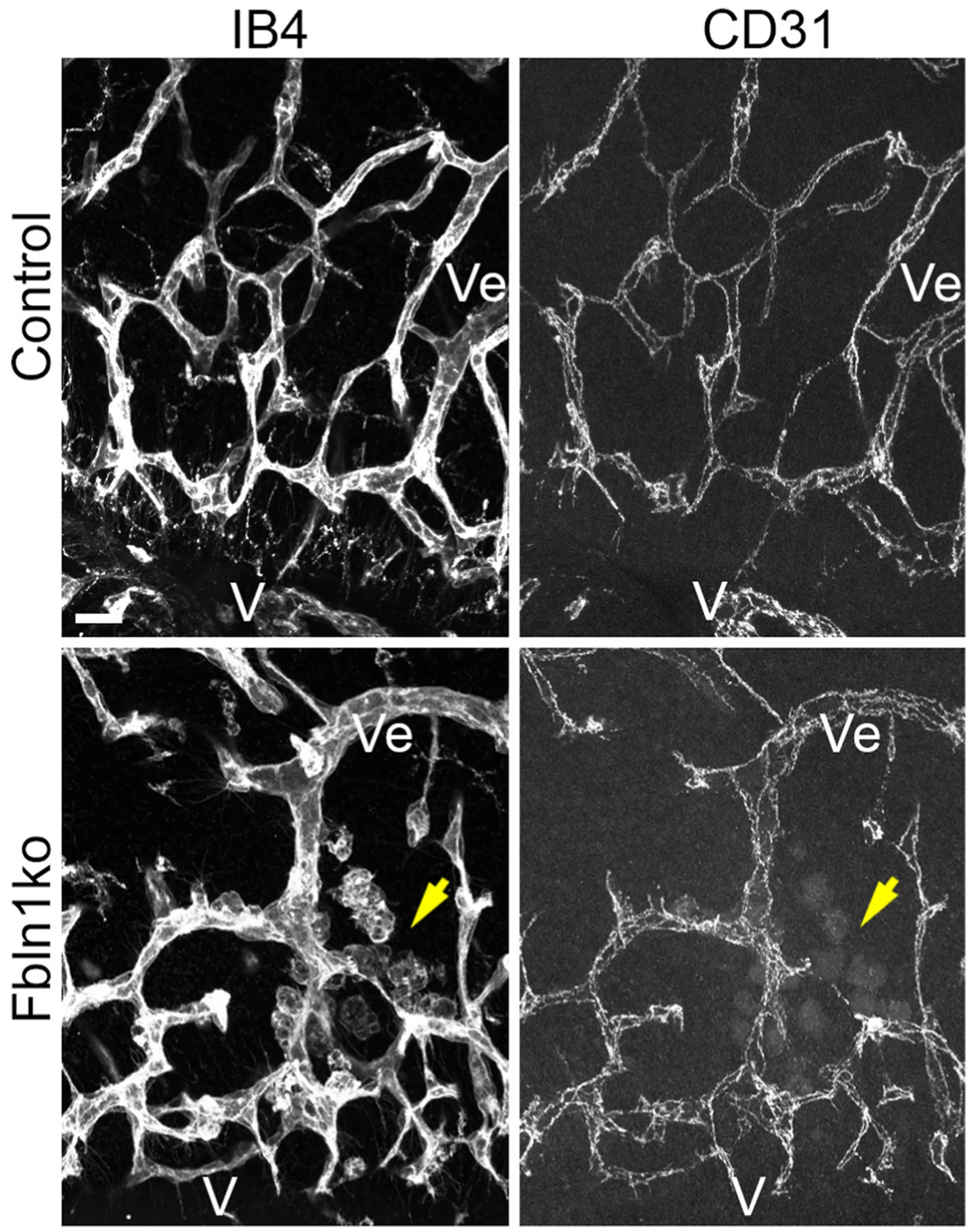
Deletion of Fbln1 disrupts venous and capillary development in the brain. Confocal images of cerebral vasculature from E17.5 control (top) and Fbln1ko (bottom) embryos stained for the indicated markers. In Fbln1 deficient brains, glomeruloid structures are associated with capillary and vein-like vessels (Ve.,Vein). These glomeruloid structures show absence or reduced CD31 staining (yellow arrow), consistent with altered endothelial cell contacts. Scale bars, 25 μm. V., Ventricle. (For interpretation of the references to colour in this figure legend, the reader is referred to the Web version of this article.)

**Fig. 10.**
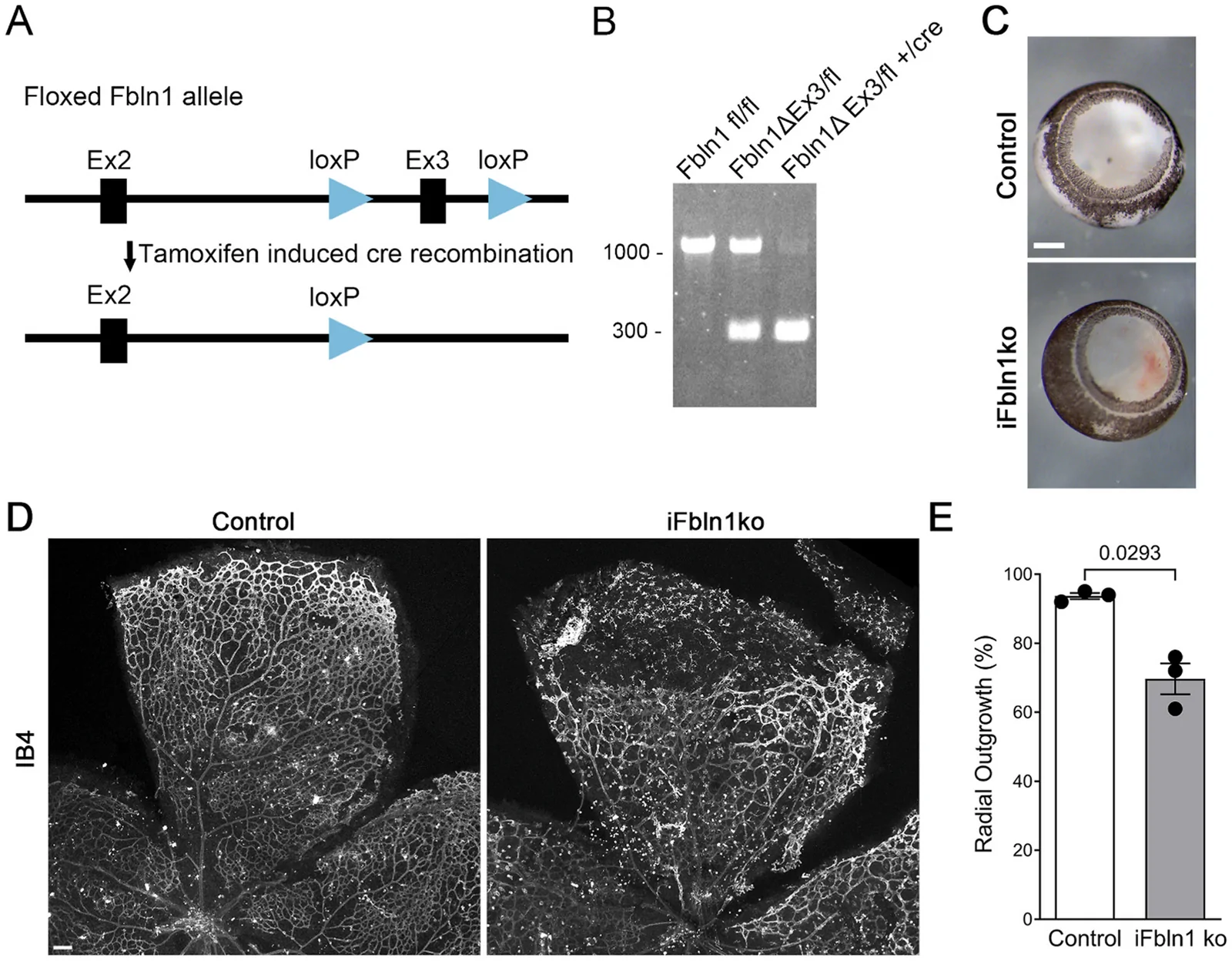
Postnatal Fbln1 deletion impairs superficial vascular plexus formation. (A) Schematic of the conditional Fbln1 allele showing loxP sites flanking exon 3 (top) and the recombined allele following tamoxifen-induced Cre-mediated deletion of exon 3 (bottom) as described in Methods section. (B) PCR genotyping of genomic DNA isolated from Fbln1 fl/fl, Fbln1 ΔEx3/fl, and Fbln1ΔEx3/fl +/cre mice showing exon 3 deletion. (C) Control and iFbln1ko pups received tamoxifen at P1, P3, and P6, and whole-mount retinas were analyzed at P8. Representative images show retinal hemorrhages in iFbln1ko mice. Scale bar, 1 mm. (D) Z-stack projections of angiogenic growth in IB4-stained P8 control and iFbln1 ko retinas. Scale bar, 100 μm. (E) Quantification of vascular outgrowth in P8 control and iFbln1ko retinas. N = 3 retinas per genotype. Black circles represent individual data points. Values are presented as mean ± SEM, and the *P* value is indicated above the bracket.

**Fig. 11.**
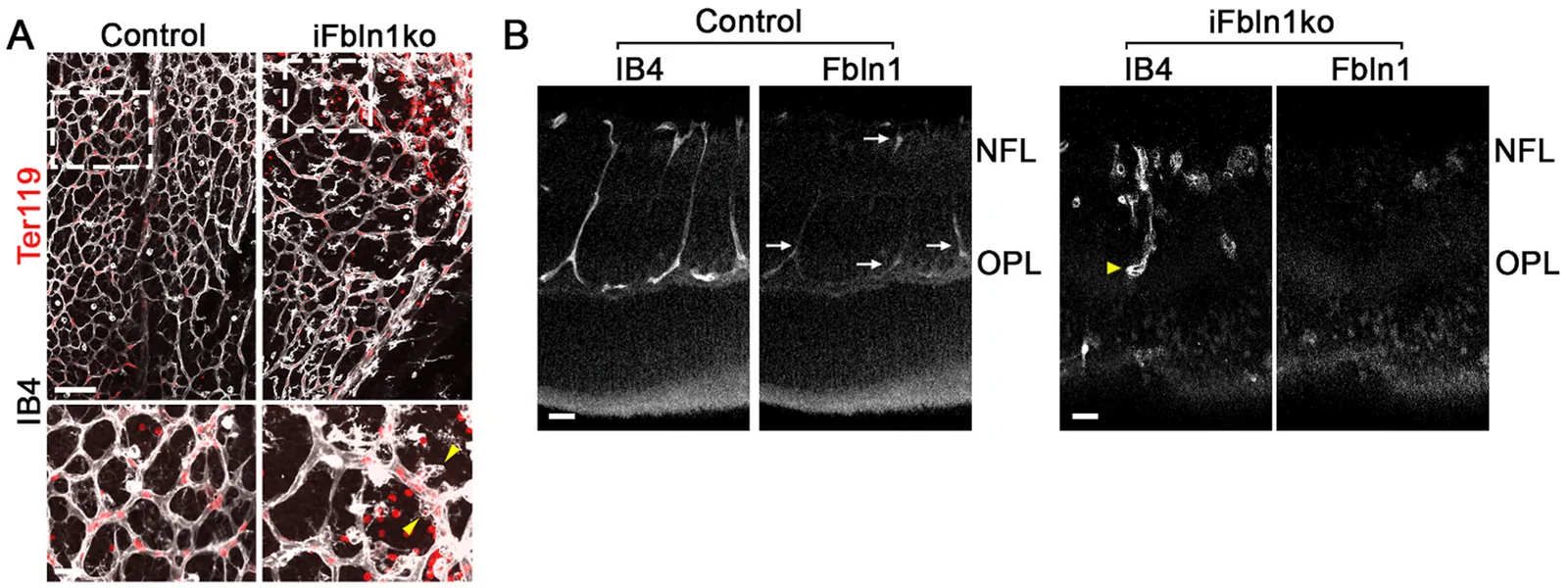
Tamoxifen-induced deletion of Fbln1 results in tortuous capillaries and glomeruloid vascular lesions in the retina. (A) Z-stack projections of the superficial vascular plexus showing glomeruloid vascular lesions and extravasated Ter119 + erythrocytes (red) in the iFbln1ko retina. Boxed regions are shown at higher magnification (lower panels), illustrating the normal vascular architecture in control retina and glomeruloid vascular malformations containing erythrocytes (yellow arrowheads) in iFbln1ko retina. Scale bar: upper panels, 100 μm; lower panels, 25 μm. (B) Immunofluorescence of vibratome retinal sections stained for isolectin B4 (IB4) and Fbln1 (antibody 2954). In control retinas, Fbln1 localizes to the basement membrane of superficial vessels and vessels extending into the inner retinal layers (white arrows), whereas Fbln1 immunoreactivity is absent from iFbln1ko vessels (yellow arrowhead) following tamoxifen-induced deletion. Scale bar, 25 μm. (For interpretation of the references to colour in this figure legend, the reader is referred to the Web version of this article.)

**Fig. 12.**
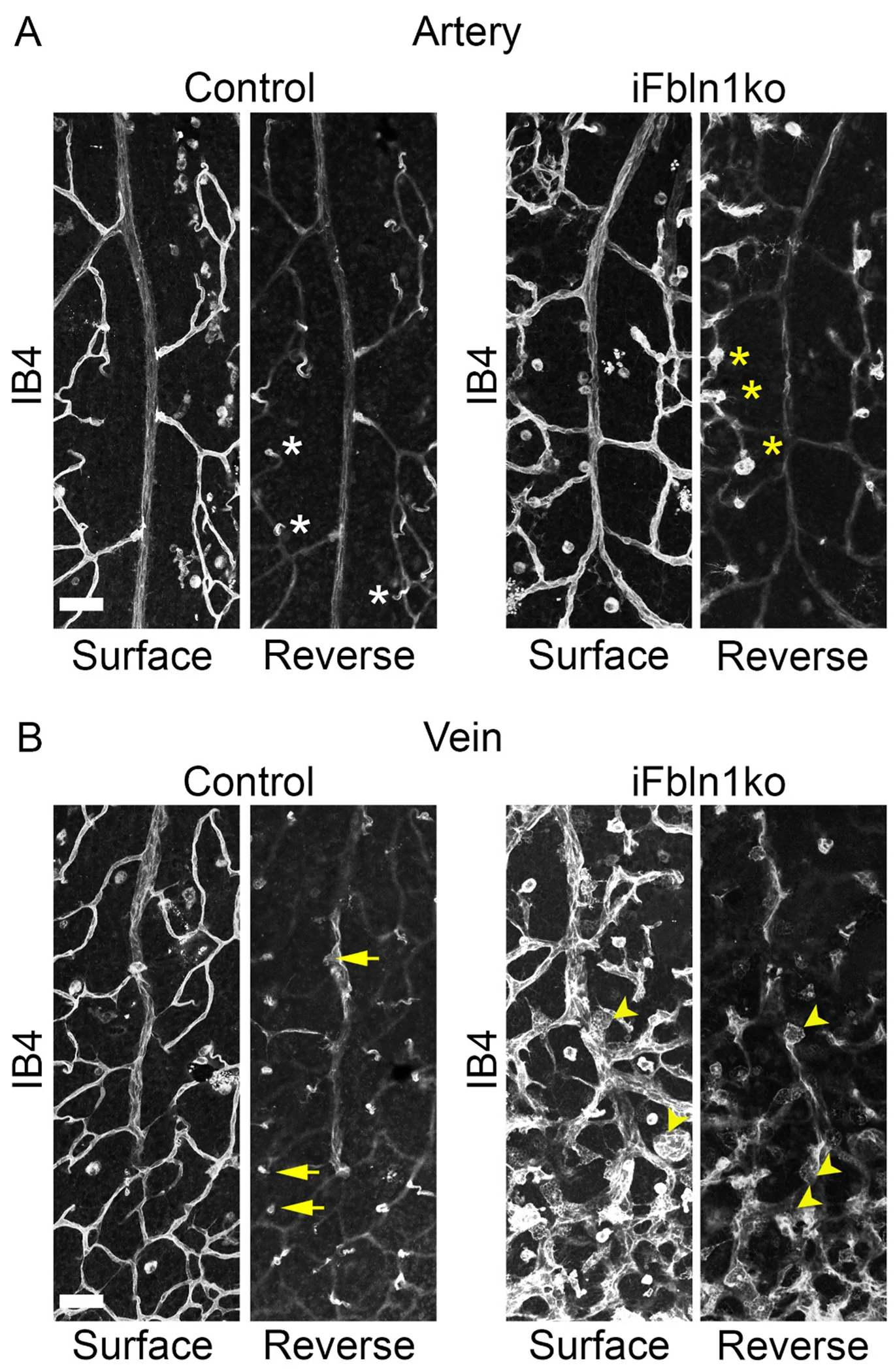
Postnatal loss of Fbln1 induces glomeruloid vascular structures associated with downward-migrating endothelial sprouts from superficial retinal veins and capillaries. Induced Fbln1 knockout (iFbln1ko) and control mice received tamoxifen at P2, P4, and P5 and retinas were analyzed at P12 with isolectin B4 (IB4). (A) Control artery and adjacent capillaries in the superficial plexus are shown in surface view and reverse side view, demonstrating normal diving EC sprouts arising from capillaries (white asterisks). In Fbln1-deficient retinas, capillaries surrounding arteries display abnormal diving EC sprouts on the reverse side of superficial vessels (yellow asterisks). (B) Control vein and adjacent capillaries in the superficial plexus are shown in surface view and reverse side view, with prominent diving EC sprouts (yellows arrows). In contrast, Fbln1-deficient veins and surrounding capillaries exhibit abnormal glomeruloid lesions associated with diving EC sprouts (yellow arrowheads, right panel, reverse view). Glomeruloid lesions are also evident in association with superficial vessels in the Fbln1-deficient retina (yellow arrowheads, left panel, surface view). Scale bar panels A and B, 50 μm. (For interpretation of the references to colour in this figure legend, the reader is referred to the Web version of this article.)

**Fig. 13.**
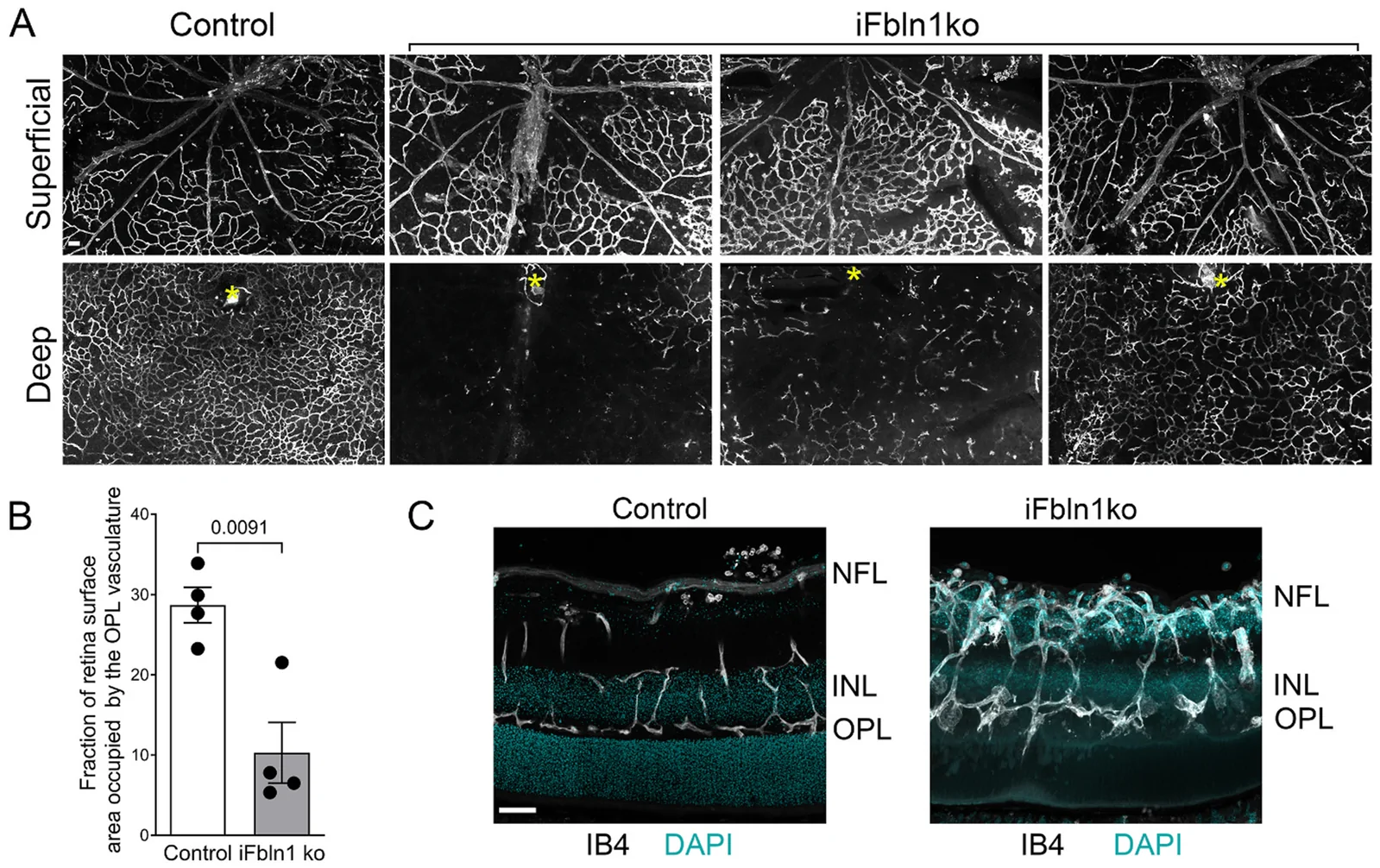
Fbln1 is required for vascularization of the deep retinal plexus. iFbln1ko and control pups received tamoxifen at P2, P4, and P5, and retinas were analyzed at P12 by IB4 staining. (A) Z-stack projections of the superficial vascular plexus (upper panels) and deep vascular plexus (lower panels). A marked reduction in deep vascular plexus formation is observed in iFbln1ko compared with controls. Yellow asterisks mark vessels associated with the optic nerve region. (B) Graph quantifying deep vascular plexus coverage as a percentage of total retinal surface area (*P* = 0.0091). N = 4 retinas per genotype. Black circles represent individual data points. Data represent mean ± SEM. (C) Z-stack projections of P12 retinal cross-sections from control and iFbln1ko mice. Note the presence of glomeruloid lesions in the deep vascular plexus of the iFbln1ko retina. Scale bar in panels A and C, 50 μm. NFL,Nerve Fiber Layer; INL, Inner Nuclear Layer; OPL, Outer Plexiform Layer. (For interpretation of the references to colour in this figure legend, the reader is referred to the Web version of this article.)

**Table 1:** Genotype frequency of offspring from mating Fbln1 +/ΔEx3 heterozygous mice and penetrance of bleeding phenotype.

Stage	Fbln1 +/+	Fbln1 +/ΔEx3	Fbln1 ΔEx3/ΔEx3	Total Mice	^a^Chi Square	Chi Square p valve	Fbln1 ΔEx3/ΔEx3 with hemorrhages	Penetrance of Bleeding Phenotype
ED11.5	7	22	11	40	1.2	0.55	0	0
ED12.5	11	21	10	42	0.05	0.98	2	20
ED13.5	14	19	9	42	1.2	0.55	9	100
ED14.5	2	7	2	11	0.8	0.66	2	100
ED16.5	4	18	9	31	2.4	0.3	9	100
ED17.5	11	10	9	26	3.6	0.17	9	100
4 weeks	20	54	0	74	26.4	p < 0.05	N/A	N/A

ED, embryonic day.

N/A, not applicable.

a Chi-squared test with critical value of 5.99 at 95% significance level.

## Data Availability

Data will be made available on request.
